# Correction: A randomized controlled trial of a combination of antiviral and nonsteroidal anti-inflammatory treatment in a bovine model of respiratory syncytial virus infection

**DOI:** 10.1371/journal.pone.0249783

**Published:** 2021-04-01

**Authors:** Paul Walsh, Maxim Lebedev, Heather McEligot, Victoria Mutua, Heejung Bang, Laurel J. Gershwin

The following information is missing from the Funding statement: This work was supported in part (H Bang) by UL1 TR001860 from the National Institutes of Health’s National Center for Advancing Translational Sciences.
