# Supplementary material for: A randomized controlled trial of a combination of antiviral and nonsteroidal anti-inflammatory treatment in a bovine model of respiratory syncytial virus infection
Source: PLoS One. 2020 Mar 12;15(3):e0230245. doi: 10.1371/journal.pone.0230245 (PMC7067438; doi:10.1371/journal.pone.0230245)
Supplement: S1 File — (DOCX) [file pone.0230245.s001.docx]

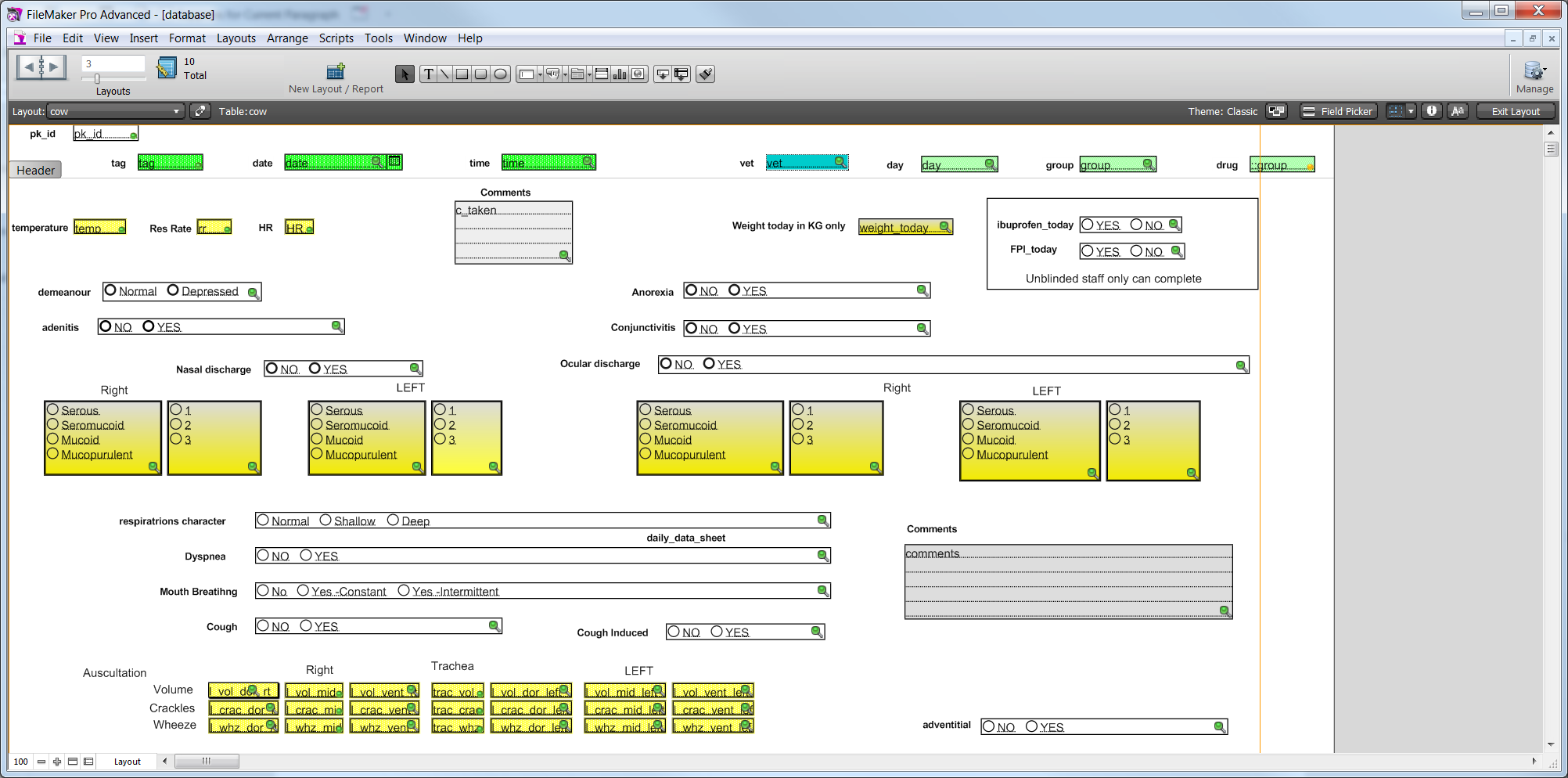


Clinical score calculated as (rectal temperature in degrees -39.5)*100 + respiratory rate +( cough*10) + (cough_induced *10) + (nasal_discharge_quantity_right *10) + (nasal_discharge_quantity_left *10) + (ocular_discharge_quantity_right *10) + (ocular_discharge_quantity_left *10) +(conjunctivitis *10) +(adenitis*10) +(adventitial *100) + (dyspnea *100) + (mouth_breathing *100) +(demeanour *100) + (anorexia*100)

Categorical variables coded as 0/1 if Yes No or present absent or by the number if ordinal as shown on the case report form
