# Supplementary material for: A randomized controlled trial of a combination of antiviral and nonsteroidal anti-inflammatory treatment in a bovine model of respiratory syncytial virus infection
Source: PLoS One. 2020 Mar 12;15(3):e0230245. doi: 10.1371/journal.pone.0230245 (PMC7067438; doi:10.1371/journal.pone.0230245)
Supplement: S3 File — (DOCX) [file pone.0230245.s003.docx]

Raw data

FPI – Antiviral Drug

Ibup Ibuprofen

Day 0 = inoculation with RSV

Day 10 = Euthanasia

Random intercept model

xtmixed collie i.ibuprofen i.fpi rcs || replicate: || new__id :

Rcs is the spline for day

predict rand_int_rep , reffects level(replicate )

predict rand_int_calf , reffects level(new__id )

graph hbar (mean) rand_int_rep ,over(replicate )

graph hbar (mean) rand_int_calf ,over(new__id )

cap drop fe_mean

cap drop replicate_effect

cap drop calf_effect

cap drop calf_mean

cap drop replicate_mean

cap drop calf_con

predict fe_mean ,xb

lab var fe_mean "FE Mean"

predict replicate_effect ,reffects level(replicate)

lab var replicate_effect "Replicate Effect"

predict calf_effect , reffects level(new__id)

lab var calf_effect "Calf Effect"

gen replicate_mean = fe_mean+ replicate_effect

gen calf_mean = fe_mean+ replicate_mean +calf_effect //+calf_con

tw (line fe_mean day ,lcolor(green) xlab(-10(1)10)) (line replicate_mean day ,lcolor(blue)) (line calf_mean day , lcolor(red) connect(ascending)) (scatter collie day if replicate ==1 , mcolor(blue) mlab(new__id))(scatter collie day if replicate ==3 , mcolor(black) mlab(new__id))(scatter collie day if replicate ==4 , mcolor(green) mlab(new__id))

Fixed Effects portion

cap drop rstand

cap drop xb

cap drop yhat

cap drop fitted

predict rstand ,rstand

predict xb , xb

predict fitted

//predict yhat , yhat

scatter xb day

scatter fitted day

scatter xb rstand

qnorm resid

qnorm rstand

scatter fitted resid

scatter replicate_effect day

scatter replicate_effect replicate_mean

scatter replicate_effect xb

estat icc

Repeating the model without calves 3_6 1_11 didn’t change the coefficients but dropping just that day, which happens to be near expected peak viral shedding

Re running the model excluding 3_6 day 5 DOES change the coefficients

. xtmixed collie i.ibuprofen i.fpi rcs if label !="3_6 5" || replicate: || new__id :

Which begs the question should I drop it?

Day by day model

The graphs look pretty much identical to the models built with just a spline function of day. (The spikes reflect the three replicates.) But trying interactions with an N this small seems a bit much.
