# Supplementary material for: A randomized controlled trial of a combination of antiviral and nonsteroidal anti-inflammatory treatment in a bovine model of respiratory syncytial virus infection
Source: PLoS One. 2020 Mar 12;15(3):e0230245. doi: 10.1371/journal.pone.0230245 (PMC7067438; doi:10.1371/journal.pone.0230245)
Supplement: S4 File — (DOCX) [file pone.0230245.s004.docx]

| **Description** | **Location in Manuscript** | **Code** | **Details** | **Interacted version** | **Alternative models (Better fit, harder to interpret) notes etc** |
| --- | --- | --- | --- | --- | --- |
|  |  |  |  |  |  |
| **Methods Table 1** | line 140 | bys replicate group :tab infect_dose if day ==0 | NA |  | NA |
|  |  |  |  |  |  |
| **Results text** |  |  |  |  |  |
| Number completing protocol | line 268 | tab euthanized_early if day ==0 | NA |  | NA |
| Number euthanized early were in replicate 1 | line 271 | bys replicate :tab euthanized_early if day ==0 | NA |  | NA |
| Number euthanzied early in each treatment group | line 270 | bys drug_treat : tab euthanized_early if day ==0 | NA |  | NA |
|  |  |  |  |  |  |
| **Table 2 Demographics** | line 275 |  |  |  |  |
|  |  | tab drug_treat if day ==0 | NA |  | NA |
|  |  | bys drug_treat : tabstat cent_wt_age1 if day ==0 ,stat(mean sd med p25 p75) | NA |  | NA |
|  |  | bys drug_treat : tab t0 if day ==0 | NA |  | NA |
|  |  | bys drug_treat :tabstat collie if day ==0, stat(p50 p25 p75 | NA |  | NA |
|  |  |  |  |  | NA |
| **Table 3**  **Results by treatment arm** |  |  |  |  |  |
|  | line 305 | xtmixed collie i.b3.drug_treat t0 cent_wt_age1 rcs if day >=0 \|\| replicate : \|\|new__id : t0 cent_wt_age1 | S5 spreadsheet | xtmixed collie i.b3.drug_treat##c.rcs t0 cent_wt_age1 rcs if day >=0\|\| replicate : \|\|new__id : t0 cent_wt_age1 | S5 drop t0/cent_weight_age1 |
|  |  | xtmixed templess_collie i.b3.drug_treat  cent_wt_age1 t0 rcs_templess if day >=0  \|\| replicate : \|\|new__id : t0 | S5 spreadsheet | xtmixed templess_collie i.b3.drug_treat##c.rcs_templess t0 cent_wt_age1 rcs if day >=0\|\| replicate : \|\|new__id : t0 | xtmixed templess_collie i.b3.drug_treat t0 cent_wt_age1 rcs if day >=0 \|\| replicate : \|\|new__id : cent_wt_age1 |
|  |  | xtmixed rr i.b3.drug_treat t0 cent_wt_age1 rsc_rr if day >=0 \|\| replicate : \|\|new__id :t0 cent_wt_age1 | S5 spreadsheet | xtmixed rr i.b3.drug_treat##c.rsc_rr t0 cent_wt_age1 rsc_rr if day >=0 \|\| replicate : \|\|new__id :t0 cent_wt_age1 | S5 drop t0/cent_weight_age1 |
|  |  | xtmixed viral_load i.b3.drug_treat rsc_v infect_dose \|\| replicate: \|\| new__id : R.infect_dose | S5 spreadsheet | Numerous alternative models with and without infecting dose of virus yield similar results | S5 drop t0/cent_weight_age1 |
| **Table 4**  **Results by treatment arm** | 335 |  |  |  |  |
| Hazards ratio for being in the top quartile of clinical score | | stcox i.b3.drug_treat t0 if day >=0 ,str(replicate ) r efron | S5 spreadsheet |  | (r = robust creates Andersen Gill estimates for multiple failure |
